# Potential Antiviral Strategy Exploiting Dependence of SARS-CoV-2 Replication on Lysosome-Based Pathway

**DOI:** 10.3390/ijms23116188

**Published:** 2022-05-31

**Authors:** Yungang Lan, Wenqi He, Gaili Wang, Zhenzhen Wang, Yuzhu Chen, Feng Gao, Deguang Song

**Affiliations:** 1 Key Laboratory of Zoonosis Research, Ministry of Education, College of Veterinary Medicine, Jilin University, Changchun 130022, China; hewq@jlu.edu.cn (W.H.); wangzhenzhen19@outlook.com (Z.W.); yuzhu_c@163.com (Y.C.); gaofeng@jlu.edu.cn (F.G.); 2 Jilin Academy of Animal Husbandry and Veterinary Medicine, Changchun 130022, China; 66xiaogai@163.com

**Keywords:** SARS-CoV-2 replication, lysosomal function, CTSL activities, lysosomal acidification

## Abstract

The recent novel coronavirus (SARS-CoV-2) disease (COVID-19) outbreak created a severe public health burden worldwide. Unfortunately, the SARS-CoV-2 variant is still spreading at an unprecedented speed in many countries and regions. There is still a lack of effective treatment for moderate and severe COVID-19 patients, due to a lack of understanding of the SARS-CoV-2 life cycle. Lysosomes, which act as “garbage disposals” for nearly all types of eukaryotic cells, were shown in numerous studies to support SARS-CoV-2 replication. Lysosome-associated pathways are required for virus entry and exit during replication. In this review, we summarize experimental evidence demonstrating a correlation between lysosomal function and SARS-CoV-2 replication, and the development of lysosomal perturbation drugs as anti-SARS-CoV-2 agents.

## 1. Introduction

Coronaviruses (CoVs), a highly diverse family of enveloped positive-sense single-stranded RNA viruses, belong to the subfamily *Orthocoronavirinae* within the family *Coronaviridae*. Four genera are found within the subfamily *Orthocoronavirinae*, namely *Alphacoronavirus*, *Betacoronavirus* (βCoV), *Gammacoronavirus*, and *Deltacoronavirus*. βCoVs exclusively infect mammalian species, leading mainly to respiratory and enteric illnesses that can be deadly [1,2]. Notably, βCoV species that can cause severe, life-threatening respiratory illnesses in humans include *Severe acute* *respiratory syndrome coronavirus* (SARS-CoV), *Middle East respiratory syndrome coronavirus* (MERS-CoV), and *Severe acute respiratory syndrome coronavirus 2* (SARS-CoV-2) [3]. To date, no prophylactic or therapeutic treatment is approved to prevent the severe illness and permanent lung damage caused by these βCoV species once the infection progresses beyond the early infection stage [4,5].

SARS-CoV-2, a member of the βCoV SARS phylogenetic cluster, is the pathogen responsible for causing the “coronavirus disease of 2019” (COVID-19) pandemic. This pandemic continues to pose insurmountable challenges to healthcare systems and healthcare professionals globally, while both the disease itself and local and national restrictions to contain the pandemic continue to directly and indirectly unleash social, health, and economic devastation on humankind. To prevent and treat this disease, more effective vaccines, drugs, and other tools are urgently needed; however, whether the use of such tools will finally put an end to COVID-19 is still debated [6,7]. On the one hand, although vaccination is underway in some countries, SARS-CoV-2 mutations and the incomplete protection afforded by vaccines (even against the unmutated virus) led to vaccine hesitancy that allowed the virus to adapt and continue to spread and cause illness. On the other hand, the United States Food and Drug Administration (FDA) approved several neutralizing antibodies, such as bamlanivimab, etesevimab, casirivimab, and imdevimab, for the treatment of hospitalized COVID-19 patients [8]. However, their extensive usage was limited by the complex intravenous administration route required, and high cost [9]. Notably, the FDA authorized Merck’s molnupiravir and Pfizer’s Paxlovid for the treatment of COVID-19 [10,11,12]. Molnupiravir, a biological prodrug of NHC (b-D-N(4)-hydroxycytidine), is a novel nucleoside analogue with a broad-spectrum antiviral activity against SARS-CoV-2 [13]. In clinical trials, molnupiravir showed beneficial effects for mild to moderate COVID-19 patients [13,14]. Paxlovid is a combination of two oral drugs: nirmatrelvir and ritonavir [15]. The FDA authorized the emergency use of Paxlovid for the treatment of mild-to-moderate COVID-19 in adults and children (12 years of age and older weighing at least 88 pounds) with a positive test for the virus that causes COVID-19, and who are at high risk for progression to severe COVID-19, including hospitalization or death [11,15]. However, as with any medication, Paxlovid and molnupiravir still have risks of more serious side effects [10,11,12]. Molnupiravir should not be taken if patients are pregnant, and Paxlovid has important safety risks for patients with transplants or kidney disease [9,11,14,16,17]. In addition, most of the approved therapeutic options, including neutralizing antibodies and oral drugs for COVID-19, are still mainly targeted at patients with mild disease, and prevent mild disease from turning into severe disease; however, there is still a lack of effective treatment for moderate and severe COVID-19 patients, which is an urgent problem to be solved clinically [9,18]. In the meantime, researchers demonstrated that the SARS-CoV-2 infection process comprised a series of steps that could be targeted to inhibit infection [19]. Nevertheless, even though several potential and promising therapeutic targets are identified to date, post-exposure antiviral therapies still need to be repeatedly optimized and upgraded to apply to people of different ages, as well as to make inexpensive, broad-spectrum anti-novel coronavirus drugs with low toxicity available.

Understanding the life cycle of SARS-CoV-2 will greatly facilitate future discoveries to guide the design of new therapeutics to treat COVID-19 [3]. Toward this goal, a series of SARS-CoV-2 studies uncovered mechanistic details of βCoV entry and replication processes in host cells. Briefly, a βCoV particle can enter a cell only after it binds to a specific cell receptor, and the subsequent fusion of viral and host cell membranes of endosomes deliver the viral genome to the cytoplasm, where it directs virus genome replication and protein synthesis prior to virion assembly. During the assembly of new virions, a portion of the endoplasmic reticulum (ER) membrane is appropriated by the virus to serve as a lipid envelope. This lipid membrane is subsequently populated with viral transmembrane proteins to package the viral genome and associated nucleocapsid proteins. However, the process by which newly assembled virions leave host cells is still unclear. The latest research shows that βCoVs exit cells by hijacking a lysosome-based pathway instead of a known biosynthetic secretory pathway commonly used by other enveloped viruses [20]. This finding is critically important, as it potentially opens up new therapeutic avenues to target lysosomotropic molecules toward inhibiting lysosomal function as a strategy to block βCoVs infection and slow virus spread.

## 2. Role of Lysosomes in SARS-CoV-2 Replication

Lysosomes, which were discovered by de Duve in the 1950s, are subcellular organelles found in nearly all types of eukaryotic cells that perform core degradative and metabolic cellular functions [21,22,23]. Lysosomes form via a budding process that pinches off membrane vesicles from the trans-Golgi network, a region of the Golgi complex responsible for sorting newly synthesized proteins [24,25]. This process generates single membrane-enclosed, spherical, dynamic, and heterogeneous organelles that vary in position, morphology, size, and contents of enzymes and substrates [13]. Importantly, lysosomes contain an acidic environment that is maintained within a low pH range of 4.5–5.5 via proton pump activity [26,27]. This low-pH environment supports activities of various hydrolytic enzymes, such as proteases, nucleases, and phosphatases that catalyze hydrolysis reactions to digest macromolecules such as proteins, nucleic acids, lipids, and carbohydrates. In addition, lysosomal fusion with endosomes or phagosomes leads to lysosomal digestion of endosome-derived small molecules and cell surface proteins or phagosome-derived large particles, such as apoptotic cell corpses and pathogenic bacteria. Furthermore, lysosomes participate in the autophagy of cytoplasmic contents, including damaged mitochondria, ER membranes, and lysosomes [28]. Lysosomes are also able to release their contents extracellularly through lysosomal exocytosis [29]. Lysosomes traffic towards the proximity of the plasma membrane (PM) along microtubules, and the lysosomal membrane and PM fuse together via a Ca^2+^-dependent process [30]. Lysosome exocytosis was shown to be important for PM wound repair by providing the additional membrane. Lysosomal secretion is commonly observed for lysosome-related organelles which exist in some specialized cell types [26,31]. In addition, the secretion of lysosomal content with regular lysosomes into extracellular space was shown in many physiological and pathological conditions [32,33].

Notably, lysosomes play key roles in host antiviral defenses through virus degradation and modulating the metabolic turnover of proteins related to immune response-associated biological signal pathways [34,35]. For example, core lysosomal proteins of human monocytic leukemia cells, including galactosidase beta 1, cathepsin A, cathepsin B, hexosaminidase subunit alpha, and hexosaminidase subunit beta, were shown to play key functional roles in cell resistance to vesicular stomatitis virus infection, an illness caused by an RNA virus [36]. As another example, Fernández de Castro et al. reported that infection with reovirus, a nonenveloped RNA virus, triggered an increase in lysosomal number and size, while also increasing lysosomal pH from ∼4.5–5 to ∼6.1 [37]. This work revealed that viral proteins are recruited to reovirus inclusions to assist in the assembly of viral components to form virions. However, before the initiation of virion assembly, modified lysosomes move toward the reovirus inclusions. Mature virions are found inside lysosomes as they are assembled and released for egress via lysosomal exocytosis, thus demonstrating that reovirus replication is a lysosome-dependent process [37]. Taken together, these and the results of other studies suggest that lysosomes may have complex (positive or negative) roles in the infection and replication processes associated with numerous viruses.

**Figure 1:**
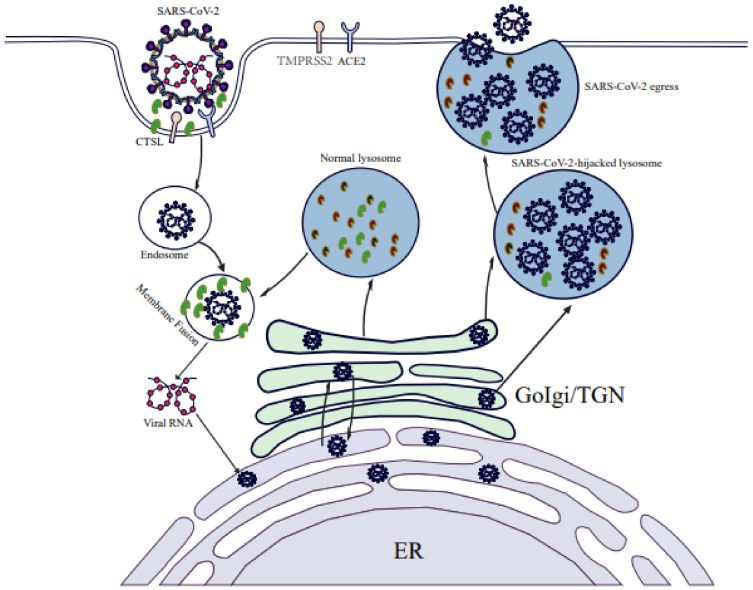
**SARS-CoV-2 replication is dependent on a lysosome-based pathway.** SARS-CoV-2 entry into the host cells is mediated by the endocytic pathway. The entry of SARS-CoV-2 and mature virus RNA release into the cytoplasm requires CTSL activity and an acid lysosomal environment. Progeny viruses exit cells by hijacking a lysosome-based pathway.

CoVs are enveloped viruses that contain the largest known viral RNA genome, which ranges in size from 26 to 32 kilobases (kb) [38]. The SARS-CoV-2 genome size is ~ 29.9 kb and shares approximately 82% sequence identity with the genome sequences of SARS-CoV and MERS-CoV, and 96.3% sequence identity with the bat CoV RaTG13 genome sequence. Comparisons of predicted protein sequences of essential enzymes and structural proteins of these viruses indicate they possess >90% identity, as determined using whole-genome sequence alignments [38,39]. Importantly, SARS-CoV-2, SARS-CoV, and MERS-CoV genomes resemble one another in that each possesses a 5′ end m7G cap structure, m7GpppA1, and a 3′-end ~ 30–60-nt-long (47-nt median length) poly-A tail that together maintain viral genome stability and prevent cellular exoribonuclease digestion. Moreover, each genome is packaged in association with viral nucleocapsid (N) proteins within a large ribonucleoprotein complex that is enclosed by an envelope membrane containing viral spike (S), envelope (E), and membrane (M) proteins. The initial steps of SARS-CoV-2 infection involve specific binding of S protein to the cellular entry receptor, namely angiotensin-converting enzyme 2 (ACE2) [40]. After initial binding of the virus to cell-surface ACE2, SARS-CoV-2 S protein is cleaved by cell-surface serine protease TMPRSS2 and cathepsin L (CTS L) to form two distinct protein fragments (S1 and S2), which are essential for S1/S2 priming, which triggers virus entry into cells via an endosome-dependent mechanism [41,42,43]. S1 is composed of an N-terminal domain, which is involved in sugar bindings, and a C-terminal domain capable of recognizing human ACE2. S2 contains the putative fusion peptide, the heptad repeats (HR1 and HR2), and is involved in the viral membrane fusion. S1 binds to ACE2 of the host cell during the virus entry, and S2 fuses, allowing the genomes to enter the host cells [44]. During the early stages of SARS-CoV-2 infection, endocytosis is followed by endosome-lysosome fusion that enables the release of SARS-CoV-2 viral RNA into the host cell cytoplasm. Indeed, different lysosomal protease activities associated with different host species were found to determine host species tropisms of various CoVs, since endosomal-lysosomal fusion is required for initiation of a complex program of viral gene expression that is highly regulated in space and time [45]. This program begins with initiation of the SARS-CoV-2 genomic replication that leads to synthesis of full-length negative-sense genomic copies that function as templates for a generation of new positive-sense copies of viral genomic RNA. In turn, these newly synthesized positive-sense genomes either: (1) engage in protein translation that generates viral nonstructural (NSP) and accessory proteins; (2) serve as replication-transcription complexes; or (3) are packaged to form new virions [3]. Open reading frames (ORFs) encoding viral structural proteins are located within the 3′-end one-third of SARS-CoV-2 genomes, while ORFs encoding accessory proteins are interspersed between these ORFs. Importantly, CoVs structural proteins participate in virion assembly and membrane budding of new virions that enable virions to leave the endoplasmic reticulum (ER) and enter the Golgi compartment. After virions enter the Golgi compartment, they can exit the cell via exocytosis to perpetuate virus transmission. Notably, recent evidence shows that SARS-CoV-2 virions preferentially exit infected cells using the lysosomal trafficking pathway after normal lysosomal pathway functions, such as lysosomal acidification, lysosomal enzyme activity, and antigen presentation, were disrupted by viral activities [20]. Taken together, results of these and numerous other studies suggest that SARS-CoV-2 replication steps, especially virus entry, virus RNA release into the cytoplasm of the cell, and progeny virus release from infected cells, are closely tied to lysosomal dysfunction (Figure 1).

## 3. SARS-CoV-2 Infection Leads to Lysosomal Dysfunction

The main function of lysosomes, to degrade or break down macromolecules, is critically important for numerous cellular processes in addition to its function as the cell’s “garbage disposal” [46]. In fact, lysosomes also act as signaling hubs that monitor intracellular levels of nutrients and energy within larger networking platforms that interconnect multiple signaling pathways related to signal transduction and autophagy regulation [47,48]. Moreover, lysosomes interact with other intracellular organelles (e.g., mitochondria, ER) that together maintain cell homeostasis [26,49,50]. Furthermore, these organelles also degrade and recycle intracellular and extracellular wastes generated during cell secretion of substances and PM repair, while also playing important roles in many other physiological and pathological processes, such as immune responses and tumorigenesis [30,50,51,52].

**Figure 2:**
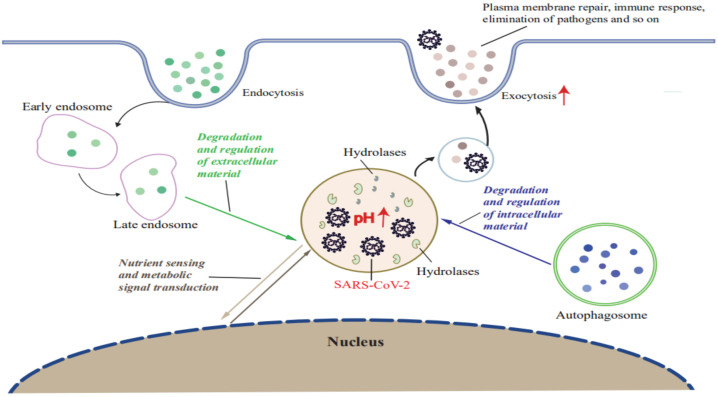
**SARS-CoV-2 infection induces lysosomal dysfunction.** SARS-CoV-2 infection leads to lysosome deacidification and promotes lysosomal exocytosis. SARS-CoV-2-hijacked lysosomes participate in the degradation and recycling of intracellular and extracellular material through autophagy and endocytosis to provide energy and source molecules. Exocytosis of SARS-CoV-2-hijacked lysosomes contributes to PM repair, the immune response, pathogen elimination, and progeny virus release in stores. SARS-CoV-2-hijacked lysosomes sense nutrients and activate metabolic signal transduction.

Indeed, for decades CoVs were detected in lysosomes during late stages of infection, although a connection between lysosomes and viral success was only proposed recently [53]. For example, Ghosh et al. recently reported results of a pioneering investigation that revealed a vital role of lysosomes in SARS-CoV-2 release. Briefly, they found that SARS-CoV-2 exploits the small Arf-like Ras family GTPase (ARL8b)-dependent lysosomal exocytosis pathway for release into the extracellular environment, and that inhibition of Rab7 GTPase, an enzyme involved in endosomal-lysosomal transport of materials, prevents egress of SARS-CoV-2 virions from cells that were associated with reduction in lysosomal number and limited endolysosomal maturation [20]. These results, when considered together with the established fact that normally the acidic environment within lysosomes helps destroy viruses and other pathogens before they leave cells, imply that SARS-CoV-2 may hijack the lysosomal acidification process by somehow promoting lysosomal deacidification that significantly weakens activities of lysosomal degradative enzymes and disrupts lysosome-dependent antigen cross-presentation [20]. Taken together, these clues support a possible scenario whereby SARS-CoV-2 infection leads to lysosomal dysfunction that lessens the ability of infected cells to degrade and recycle intracellular and extracellular materials through autophagy and endocytosis (Figure 2). In turn, virus-induced lysosomal dysfunction interferes with other cellular functions, such as PM repair, the immune response, signal transduction, pathogen elimination, and so on (Figure 2). As a result, SARS-CoV-2 virions that exit cells remain intact and ready to infect other cells to begin a new infection cycle. In addition, deacidification of SARS-CoV-2-hijacked lysosomes may alter immune system functions to account for observed COVID-19-associated immune system abnormalities [20,51].

Lysosomal exocytosis results in the PM localization of lysosomal membrane proteins and also the release of lysosomal contents into the extracellular environment [54,55]. ARL8b localizes to late endosomes/lysosomes and regulates their movement to the PM and, ultimately, their exocytosis. During SARS-CoV-2-hijacking lysosome exocytosis, the multisubunit complex (BORC) recruiting ARL8b (BORC-ARL8b) complex drives the anterograde transport of lysosomes from perinuclear regions to the vicinity of the PM [20]. Fusion of lysosomes with the PM is mediated by the soluble N-ethylmaleimide-sensitive factor attachment protein receptor (SNARE) complex composed of VAMP7, STX4, and SNAP23, and also requires an increase in the intracellular and/or localized Ca^2+^ level. Chen et al. showed that SARS-CoV-2 infection promoted lysosomal exocytosis (Figure 2) and revealed a mechanism by which SARS-CoV-2 interacts with host factors to promote its extracellular egress [43]. Briefly, SARS-CoV-2 NSP (ORF3a) facilitates lysosomal targeting of the BORC-ARL8b complex, which mediates the trafficking of lysosomes to the vicinity of the PM, and exocytosis-related SNARE proteins [56]. The Ca^2+^ channel TRPML3 is required for SARS-CoV-2 ORF3a-mediated lysosomal exocytosis [56]. Ser171 and Trp193 in SARS-CoV-2 ORF3a are critical for promoting lysosomal exocytosis and blocking autophagy [56]. However, further research is needed to shed more light on the role of ORF3a in the lysosomal exocytosis-mediated egress of SARS-CoV-2.

Lysosomal degradation of materials is tightly regulated by pH and hydrolase activities. The basal lysosomal pH range of 4.5–5.0 is associated with a buffering capacity of 19 ± 6 mM/pH unit [57]. Each lysosome consists of an outer membrane that surrounds an interior acidic environment. The low internal pH is maintained via proton pumps acting through multiple types of membrane channels that include chloride channels (e.g., CLC-7, CLC-6, CLC-3), calcium channels (e.g., TRPML1), and vacuolar H^+^-ATPase (V-ATPase) [58]. Meanwhile, lysosomes contain numerous hydrolases, which are synthesized within the ER followed by entry into lysosomes via at least two main mechanisms: one mechanism mainly involves modification of hydrolytic enzymes through the addition of mannose 6-phosphate receptor (M6PR) residues that are recognized by the Golgi apparatus then transported to the lysosome; the other mechanism involves transport by lysosomal integral membrane protein 2 (LIMP-2) of hydrolases such as glucocerebrosidases into lysosomes [59,60]. Normal lysosomal degradative functions are important for cellular functions, as dysregulated lysosomal degradation can lead to impaired endocytic function, autophagic degradation, and macromolecule biogenesis and transport that are associated with proteinopathic neurodegenerative diseases, metabolic disorders, and immunological diseases [61,62,63]. In addition, lysosomal degradation dysfunction can disrupt functions of other organelles (e.g., mitochondria), leading to increased production of reactive oxygen species and inflammatory cytokines that can contribute to pathogenesis of inflammatory diseases, cancer, and infectious diseases [64,65,66].

Remarkably, host cell fusion with βCoV particles, which occurs after virions bind to their respective host cell receptors, is induced by lysosomal hydrolases (e.g., CTSL) that are sensitive to lysosomal pH [3,43]. After fusion is complete, βCoV viral particles enter the endolysosomal intracellular transport system, where the low-pH environment supports cleavage of viral proteins and subsequent release of viral RNA into the cytoplasm, and viral exit from cells is due to viral hijacking of a lysosome-based pathway [3,7,30]. Although SARS-CoV-2 replication depends on altered lysosomal pH and verified roles of host cell hydrolases, it is still unclear whether lysosomal deacidification of cells is actually caused by SARS-CoV-2 infection [67]. Alternatively, it was postulated that infected cells may actively deacidify lysosomes to promote the release of newly generated SARS-CoV-2 from cells as a mechanism for relieving viral pressure within infected cells [67]. Consequently, researchers speculate that lysosomal deacidification may be a consequence of the action of specific SARS-CoV-2 proteins, while other researchers suggest that lysosomal deacidification is indirectly caused by too much cargo loading and/or disruption of proton pump or ion channel trafficking functions [67,68,69]. In short, it is necessary to explore the mechanism underlying SARS-CoV-2 infection-associated lysosome deacidification in order to devise strategies to re-acidify lysosomes so they can destroy viruses, inhibit virus egress, and restore antigen-presenting cell (APC) function [67].

## 4. Targeting Lysosome-Based Replication of SARS-CoV-2 as a Potential Antiviral Strategy for Controlling COVID-19 Outbreaks

Cell type and environmental conditions influence lysosomal function and functions of associated cellular pathways (e.g., autophagy, endocytosis) [69]. In turn, these effects can trigger pathological effects stemming from lysosome dysfunction-associated gene mutations or accumulated proteins that were detected in cells of individuals suffering from lysosomal storage disorders, neurodegeneration, infectious diseases, cancers, and aging [58,59,60,61,62,63]. Thus, biomaterials research strategies for restoring lysosomal function may potentially improve human health. In the case of COVID-19, due to the continual creation of new mutations within SARS-CoV-2 genomes, a worthwhile strategy for designing SARS-CoV-2 antiviral drugs might entail blocking of lysosome-based replication of SARS-CoV-2 instead of inhibiting activities of specific key viral proteins required for SARS-CoV-2 infection.

Based on the role of lysosomes in SARS-CoV-2 replication, targeting lysosomal hydrolase activity, pH, biogenesis, and exocytosis may be prevention strategies for COVID-19. When considering lysosomes as targets, it is important to note the need for specificity; that is, agents that will not target all lysosomes, but will specifically target those lysosomal proteins that are defective in certain organs, tissues, or cells [46]. On the other hand, both the formation of mature lysosomes and lysosomal exocytosis are complex processes. The biogenesis of lysosomes involves the fusion of late endosomes that contain material taken up at the cell surface with transport vesicles that bud from the trans-Golgi network, and lysosomal exocytosis involves the luminal content of the lysosome in the extracellular milieu [26,70,71]. Thus, targeting lysosomal formation and exocytosis may lead to unacceptable side effects because of the integral role of lysosomes in several key physiological processes [58,59,60,61,62,63]. At present, COVID-19 drug research hotspots focus on modulating lysosomal hydrolase activity and acidification. The antiviral effects of the compounds within our manuscript were summarized in Supplementary File S1.

### 4.1. Using CTSL Inhibitors

CTSL, a member of the lysosomal cysteine protease family, contains an L domain structure consisting mainly of α-helix and an R domain structure consisting mainly of β-sheet [72]. The availability of various selective CTSL inhibitors offers opportunities to both block SARS-CoV-2 entry into human host cells and inhibit viral RNA cytoplasmic release and the initiation of virus replication [73]. Thus, ready-to-use CTSL inhibitors should be explored as treatment options for COVID-19. One such inhibitor, amantadine, a licensed anti-influenza drug, was shown to significantly inhibit CTSL activity after SARS-CoV-2 pseudovirus infection by suppressing transcription of the CTSL-encoding gene to prevent SARS-CoV-2 infection both in vitro and in vivo [74,75,76]. Another CTSL inhibitor, teicoplanin, is a glycopeptide antibiotic that appears to inhibit SARS-CoV-2 infection by inhibiting CTSL activity [77], while astaxanthin, a potential immunomodulatory antioxidant agent, was shown to suppress CTSL activity in both Syrian hamster embryo cells and muscle cells [5,78,79]. Meanwhile, numerous other potential CTSL inhibitors were discovered within the FDA-approved drug inventory database that may effectively block SARS-CoV-2 infection, as summarized in earlier reviews [73,80]. In addition, several Chinese medicinal extracts that are currently in widespread use for treating patients with SARS-CoV-2 infections were shown to inhibit CTSL activity, although they have not yet received US FDA approval. For example, MOL736, also known as aurantiamide acetate derived from Artemisia annua L. plant, was shown to inhibit CTSL activity, relieve cough, and reduce sputum production [81]. Moreover, water and ethanol extracts of Drynariae Rhizoma were also shown to possess significant CTSL inhibitory activities [73,82]. Nevertheless, achieving therapeutically effective and safe CTSL inhibitor blood levels using various drug administration routes can sometimes be challenging, due to the risk of severe adverse effects and/or (in this case) undesirable (intrinsic) pharmacological effects. Thus, although several CTSL inhibitors were shown to interfere with SARS-CoV-2 infection, further research is needed to shed light on how best to administer these inhibitors in clinical settings in order to maximize their safety and effectiveness as post-exposure treatments for SARS-CoV-2 infections.

### 4.2. Modulating Lysosomal Acidification

**Figure 3:**
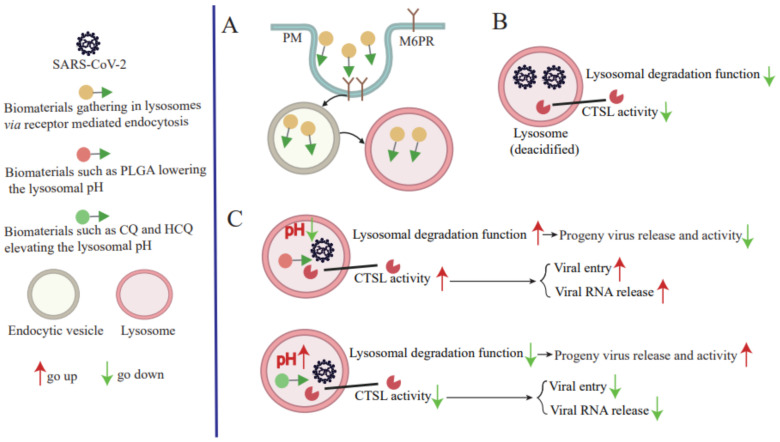
**SARS-CoV-2 replication can be inhibited by modulating lysosomal pH.** (**A**) Lysosomotropic compounds allow for lysosomal localization via receptor-mediated endocytosis. (**B**) SARS-CoV-2 infection leads to lysosome deacidification. (**C**) The antiviral agent for SARS-CoV-2 is a complex process of lysosomal pH modulation.

According to accumulated knowledge regarding the relationship between lysosomal function and SARS-CoV-2 infections, essential requirements for SARS-CoV-2 replication include (1) CTSL activity, which facilitates initial mature virus entry and RNA release into the cytoplasm and requires an acid lysosomal environment; and (2) lysosome deacidification, which is important for progeny virus release from cells to generate infectious virus to initiate new rounds of infection. Taken together, these findings suggest that lysosomes have a complex (positive or negative) role in SARS-CoV-2 infection or replication processes, with an absence of lysosomal acidification blocking SARS-CoV-2 replication. Thus, modulating lysosomal acidification or pH using treatments that induce changes in CTSL activity and lysosomal degradation function may make it possible to block the SARS-CoV-2 infection cycle (Figure 3).

Lysosomotropic compounds, which are small molecules that selectively accumulate within lysosomes regardless of their chemical nature or mechanism of uptake, are always associated with intrinsic pharmacological functions [83]. Importantly, several lysosomotropic molecules with basic pH characteristics are known to increase lysosomal pH during degradative processes, while those with acidic characteristics are known to decrease lysosomal pH (Figure 3A) [27]. Therefore, in order to modulate lysosomal pH, lysosomotropic molecules released by enzymatic cleavage of molecules within the acidic lysosomal environment should be either bases or acids. More specifically, the pKa of the released component should exceed the basal lysosomal pH value (4.5–5.0) to elevate lysosomal pH, or should be less than the basal pH value in order to reduce lysosomal pH.

Lysosomotropic chloroquine (CQ) (pKa values of 4.0, 8.4, and 10.2), an older FDA approved antimalarial drug, and its better-tolerated analog hydroxychloroquine (HCQ) (pKa values of 4.0, 8.3, and 9.7), may inhibit SARS-CoV-2 infection by releasing basic side chains that raise lysosomal pH [84,85]. In fact, CQ/HCQ-induced pH elevation was shown to suppress SARS-CoV-2 entry and viral RNA release by decreasing CTSL activity, while also disrupting COVID-19-associated lysosomal autophagic processes by suppressing lysosomal degradative functions (Figure 3C). Nonetheless, use of CQ for the treatment of COVID-19 triggered significant debate, especially since the drug is associated with side effects and exhibits only marginal efficacy [86,87,88]. Meanwhile, the lysosomotropic-alkalizing molecules ROC-325 (with a pKa 8.3) may be an alternative lysosome-based drug repurposing opportunity for COVID-19 treatment [89]. Moreover, other lysosomotropic molecules such as Nitazoxanide (pKa 8.3), IITZ-01 (pKas 4.7, 5.4, 11.54, 12.54 13.7, 54.88), IITZ-02 (pKa 4.75, 5.42, 11.56, 12.65, 14.49), and obatoclax mesylate (pKas 4.68, 13.97) can elevate lysosomal pH by releasing basic groups, warranting further study for COIVD-19 treatment [27,57,90,91].

With regard to alternative approaches for achieving lysosomal deacidification, the macrolide antibiotic bafilomycin A1, a lysosomal V-ATPase inhibitor that targets the V-ATPase ATP6V0C/V0 subunit c, inhibits lysosomal acidification by preventing protons from passing into the lysosomal lumen (Figure 3C) [92]. Meanwhile, another agent that possesses a lysosome pH-neutralizing function, ammonium chloride (NH_4_Cl), can also prevent lysosome acidification [93,94]. In fact, endosomal acidification inhibitors bafilomycin A1 and NH_4_Cl were shown to exert antiviral effects against SARS-CoV-2 in vitro cell models and in vivo in hACE2 transgenic mice, and thus should be evaluated as potential COVID-19 treatments [95].

As outlined above, regardless of their pharmacological effects, accumulating evidence suggests that raising the lysosomal pH from 4.5–5 to 6–6.5, a pH value above the pH optimum of CTSL and pH optima of most other lysosomal enzymes, may inhibit SARS-CoV-2 infection [5,27,88]. However, compounds such as CQ and bafilomycin A1 may actually support progeny virus release, which requires lysosome deacidification, thus offsetting the antiviral effects of these agents. Importantly, although substantially fewer known compounds and materials lower lysosomal pH than increase lysosomal pH, lysosomal pH-lowering compounds should be assessed for anti-SARS-CoV-2 effects. Such agents include polyester and poly (lactic-co-glycolic acid) (PLGA), the latter of which was approved by the US FDA for biomedical and pharmaceutical applications (Figure 3C). PLGA degrades in an acidic environment to release component lactic and glycolic carboxylic acids with pKa values of 3.86 and 3.83, respectively [96]. Moreover, this agent was shown to reduce lysosomal pH in models of various diseases associated with elevated lysosomal pH and is the top polymeric candidate agent for use in the fabrication of drug delivery devices [96,97,98]. Notably, PLGA-encapsulated curcumin nanoparticles were shown in vitro to possess anti-COVID-19 activities, although it is unclear whether PLGA directly inhibits virus replication by enhancing lysosome degradation of the virus or acts via a different mechanism [99]. 

## 5. Conclusions

This review summarizes the correlation between lysosomes and virus replication by describing multiple roles played by lysosomes during SARS-CoV-2 replication and by providing potential mechanisms to explain how SARS-CoV-2 infection leads to lysosomal dysfunction. However, the detailed mechanisms underlying virus-induced lysosome dysfunction must be explored further through investigations of SARS-CoV-2-induced active and passive lysosome deacidification mechanisms within infected cells, as must the effects of lysosomal reacidification on SARS-CoV-2 egress and antigen presentation in APCs. Moreover, here the significance of CTSL activity and lysosomal pH modulation in COVID-19 disease are also discussed, with a focus on exploiting lysosomotropic molecules for use as anti-SARS-CoV-2 agents that act to specifically modulate lysosomal pH. Nevertheless, any strategy based on the alteration of lysosomal pH must take into account the different roles played by lysosomal acidification and deacidification during different stages of viral replication before the therapeutic potential of lysosome pH-modulating agents can be tapped to guide the development of clinical treatments to block virus infection.

In addition, the novel oral anti-SARS-CoV-2 drugs are potentially immense and may bring new hope for COVID-19 treatment and recovery [18]. Recently, the oral antivirals of COVID-19 were shown to generally function against viral enzyme structures such as RNA-dependent RNA polymerase (RdRp), main protease (3CLpro), and papain-like protease (PLpro), which are important for viral replication [8,9,18]. Paxlovid targets the SARS-CoV-2 main protease referred to as 3CLpro or nsp5 protease [11]. Molnupiravir is used as a competitive alternative substrate and targets SARS-CoV-2 RdRp [10]. Thus, both oral antivirals can prevent the SARS-CoV-2 virus from copying itself directly. Although they are less affected by SARS-CoV-2 mutation, due to the continual occurrence of mutations of SARS-CoV-2 genomes, a worthwhile strategy for designing SARS-CoV-2 antiviral drugs might be to block a general cellular mechanism involved in the virus replication process instead of inhibiting specific viral proteins crucially needed for SARS-CoV-2 infection. For example, based on lysosome roles in SARS-CoV-2 replication, targeting lysosome-based replication of SARS-CoV-2 may be a prevention strategy for COVID-19, as it affects virus entry, virus RNA release, and progeny virus release. Interestingly, whether the oral drugs for treatment of COVID-19, such as Paxlovid and molnupiravir, are lysosomotropic molecularly, and can indirectly affect the anti-SARS-CoV-2 effects by modulating lysosomal function, warrants further study.

## Data Availability

Not applicable.

## Supplementary Materials

The following supporting information can be downloaded at: https://www.mdpi.com/article/10.3390/ijms23116188/s1.

## Abbreviations

CoVs	Coronaviruses
βCoV	Betacoronavirus
COVID-19	Coronavirus disease 2019
SARS-CoV	Severe acute respiratory syndrome coronavirus
MERS-CoV	Middle East respiratory syndrome coronavirus
SARS-CoV-2	Severe acute respiratory syndrome coronavirus 2
CTSL	Cathepsin L
ACE2	Angiotensin-converting enzyme 2
PM	Plasma membrane
V-ATPase	Vacuolar H^+^-ATPase
CQ	Chloroquine
HCQ	Hydroxychloroquine
APC	Antigen-presenting cell
PLGA	Poly (lactic-co-glycolic acid)
FDA	The United States Food and Drug Administration
ORFs	Open reading frames

## References

[1] Corman V.M., Muth D., Niemeyer D., Drosten C. (2018). Hosts and Sources of Endemic Human Coronaviruses. Adv. Virus Res..

[2] Paules C.I., Marston H.D., Fauci A.S. (2020). Coronavirus Infections-More Than Just the Common Cold. JAMA.

[3] V’Kovski P., Kratzel A., Steiner S., Stalder H., Thiel V. (2021). Coronavirus biology and replication: Implications for SARS-CoV-2. Nat. Rev. Microbiol..

[4] Napoli C., Benincasa G., Criscuolo C., Faenza M., Liberato C., Rusciano M. (2021). Immune reactivity during COVID-19: Implications for treatment. Immunol. Lett..

[5] Blaess M., Kaiser L., Sauer M., Csuk R., Deigner H.P. (2020). COVID-19/SARS-CoV-2 Infection: Lysosomes and Lysosomotropism Implicate New Treatment Strategies and Personal Risks. Int. J. Mol. Sci..

[6] Ahamed F., Ganesan S., James A., Zaher W.A. (2021). Understanding perception and acceptance of Sinopharm vaccine and vaccination against COVID-19 in the UAE. BMC Public Health.

[7] Singh L., Bansal S., Bode L., Budak C., Chi G., Kawintiranon K., Padden C., Vanarsdall R., Vraga E., Wang Y. (2020). A first look at COVID-19 information and misinformation sharing on Twitter. arXiv.

[8] Kumar S., Chandele A., Sharma A. (2021). Current status of therapeutic monoclonal antibodies against SARS-CoV-2. PLoS Pathog..

[9] Kim S. (2022). COVID-19 Drug Development. J. Microbiol. Biotechnol..

[10] (2021). Merck and Ridgeback’s Investigational Oral Antiviral Molnupiravir Reduced the Risk of Hospitalization or Death by Approximately 50 Percent Compared to Placebo for Patients with Mild or Moderate COVID-19 in Positive Interim Analysis of Phase 3 Study—Merck.com. https://www.merck.com/news/.

[11] COVID-19 Antiviral Efforts | Pfizer. https://www.pfizer.com/science/coronavirus/antiviral-efforts.

[12] Food and Druga Administration (FDA) Emergency Use Authorization 105. 22 December 2021. Paxlovid (Nirmatrelvir Co-Packaged with Ritonavir) for the Treatment of Mild-to-Moderate Coronavirus Disease 2019 (COVID-19) in Certain Adults and Pediatric Patients. https://www.fda.gov/media/155049/download.

[13] Menendez-Arias L. (2021). Decoding molnupiravir-induced mutagenesis in SARS-CoV-2. J. Biol. Chem..

[14] Mahase E. (2021). COVID-19: Molnupiravir reduces risk of hospital admission or death by 50% in patients at risk, MSD reports. BMJ.

[15] Pfizer’s Novel COVID-19 Oral Antiviral Treatment Candidate Reduced Risk of Hospitalization or Death by 89% in Interim Analysis of Phase 2/3 EPIC-HR Study. https://www.pfizer.com/news/pressrelease/press-release-detail.

[16] Fishbane S., Hirsch J.S., Nair V. (2022). Special Considerations for Paxlovid Treatment Among Transplant Recipients with SARS-CoV-2 Infection. Am. J. Kidney Dis..

[17] Tian L., Pang Z., Li M., Lou F., An X., Zhu S., Song L., Tong Y., Fan H., Fan J. (2022). Molnupiravir and Its Antiviral Activity Against COVID-19. Front. Immunol..

[18] Wen W., Chen C., Tang J., Wang C., Zhou M., Cheng Y., Zhou X., Wu Q., Zhang X., Feng Z. (2022). Efficacy and safety of three new oral antiviral treatment (molnupiravir, fluvoxamine and Paxlovid) for COVID-19: A meta-analysis. Ann. Med..

[19] Zhou Y.W., Xie Y., Tang L.S., Pu D., Zhu Y.J., Liu J.Y., Ma X.L. (2021). Therapeutic targets and interventional strategies in COVID-19: Mechanisms and clinical studies. Signal Transduct. Target. Ther..

[20] Ghosh S., Dellibovi-Ragheb T.A., Kerviel A., Pak E., Qiu Q., Fisher M., Takvorian P.M., Bleck C., Hsu V.W., Fehr A.R. (2020). βCoronaviruses Use Lysosomes for Egress Instead of the Biosynthetic Secretory Pathway. Cell.

[21] Eichel H.J., Bukovsky J. (1961). Intracellular distribution pattern of rat liver glutamic-oxalacetic transaminase. Nature.

[22] Saftig P., Klumperman J. (2009). Lysosome biogenesis and lysosomal membrane proteins: Trafficking meets function. Nat. Rev. Mol. Cell Biol..

[23] De Duve C. (2005). The lysosome turns fifty. Nat. Cell Biol..

[24] Braulke T., Bonifacino J.S. (2009). Sorting of lysosomal proteins. Biochim. Biophys. Acta.

[25] Luzio J.P., Hackmann Y., Dieckmann N.M., Griffiths G.M. (2014). The biogenesis of lysosomes and lysosome-related organelles. Cold Spring Harb. Perspect. Biol..

[26] Yang C., Wang X. (2021). Lysosome biogenesis: Regulation and functions. J. Cell Biol..

[27] Zeng J., Shirihai O.S., Grinstaff M.W. (2020). Modulating lysosomal pH: A molecular and nanoscale materials design perspective. J. Life Sci..

[28] Bright N.A., Davis L.J., Luzio J.P. (2016). Endolysosomes Are the Principal Intracellular Sites of Acid Hydrolase Activity. Curr. Biol..

[29] Tancini B., Buratta S., Delo F., Sagini K., Chiaradia E., Pellegrino R.M., Emiliani C., Urbanelli L. (2020). Lysosomal Exocytosis: The Extracellular Role of an Intracellular Organelle. Membranes.

[30] Medina D.L., Fraldi A., Bouche V., Annunziata F., Mansueto G., Spampanato C., Puri C., Pignata A., Martina J.A., Sardiello M. (2011). Transcriptional activation of lysosomal exocytosis promotes cellular clearance. Dev. Cell.

[31] Samie M., Wang X., Zhang X., Goschka A., Li X., Cheng X., Gregg E., Azar M., Zhuo Y., Garrity A.G. (2013). A TRP channel in the lysosome regulates large particle phagocytosis via focal exocytosis. Dev. Cell.

[32] Maeda F.Y., van Haaren J.J., Langley D.B., Christ D., Andrews N.W., Song W. (2021). Surface-associated antigen induces permeabilization of primary mouse B-cells and lysosome exocytosis facilitating antigen uptake and presentation to T-cells. Elife.

[33] Westman J., Plumb J., Licht A., Yang M., Allert S., Naglik J.R., Hube B., Grinstein S., Maxson M.E. (2022). Calcium-dependent ESCRT recruitment and lysosome exocytosis maintain epithelial integrity during *Candida albicans* invasion. Cell Rep..

[34] Choi Y., Bowman J.W., Jung J.U. (2018). Autophagy during viral infection—A double-edged sword. Nat. Rev. Microbiol..

[35] Castrejon-Jimenez N.S., Leyva-Paredes K., Hernandez-Gonzalez J.C., Luna-Herrera J., Garcia-Perez B.E. (2015). The role of autophagy in bacterial infections. Biosci. Trends.

[36] Xu B., Gao Y., Zhan S., Ge W. (2017). Quantitative proteomic profiling for clarification of the crucial roles of lysosomes in microbial infections. Mol. Immunol..

[37] Fernandez de Castro I., Tenorio R., Ortega-Gonzalez P., Knowlton J.J., Zamora P.F., Lee C.H., Fernandez J.J., Dermody T.S., Risco C. (2020). A modified lysosomal organelle mediates nonlytic egress of reovirus. J. Cell Biol..

[38] Lu R., Zhao X., Li J., Niu P., Yang B., Wu H., Wang W., Song H., Huang B., Zhu N. (2020). Genomic characterisation and epidemiology of 2019 novel coronavirus: Implications for virus origins and receptor binding. Lancet.

[39] Naqvi A.A.T., Fatima K., Mohammad T., Fatima U., Singh I.K., Singh A., Atif S.M., Hariprasad G., Hasan G.M., Hassan M.I. (2020). Insights into SARS-CoV-2 genome, structure, evolution, pathogenesis and therapies: Structural genomics approach. Biochim. Biophys. Acta Mol. Basis Dis..

[40] Shang J., Ye G., Shi K., Wan Y., Luo C., Aihara H., Geng Q., Auerbach A., Li F. (2020). Structural basis of receptor recognition by SARS-CoV-2. Nature.

[41] Abbasi A.Z., Kiyani D.A., Hamid S.M., Saalim M., Fahim A., Jalal N. (2021). Spiking dependence of SARS-CoV-2 pathogenicity on TMPRSS2. J. Med. Virol..

[42] Lei C., Qian K., Li T., Zhang S., Fu W., Ding M., Hu S. (2020). Neutralization of SARS-CoV-2 spike pseudotyped virus by recombinant ACE2-Ig. Nat. Commun..

[43] Zhao M.M., Yang W.L., Yang F.Y., Zhang L., Huang W.J., Hou W., Fan C.F., Jin R.H., Feng Y.M., Wang Y.C. (2021). Cathepsin L plays a key role in SARS-CoV-2 infection in humans and humanized mice and is a promising target for new drug development. Signal Transduct. Target. Ther..

[44] Li F. (2016). Structure, Function, and Evolution of Coronavirus Spike Proteins. Annu. Rev. Virol..

[45] Zheng Y., Shang J., Yang Y., Liu C., Wan Y., Geng Q., Wang M., Baric R., Li F. (2018). Lysosomal Proteases Are a Determinant of Coronavirus Tropism. J. Virol..

[46] Bonam S.R., Wang F., Muller S. (2019). Lysosomes as a therapeutic target. Nat. Rev. Drug Discov..

[47] Maxfield F.R. (2014). Role of endosomes and lysosomes in human disease. Cold Spring Harb. Perspect. Biol..

[48] Savini M., Zhao Q., Wang M.C. (2019). Lysosomes: Signaling Hubs for Metabolic Sensing and Longevity. Trends Cell Biol..

[49] Cheng H., Gang X., He G., Liu Y., Wang Y., Zhao X., Wang G. (2020). The Molecular Mechanisms Underlying Mitochondria-Associated Endoplasmic Reticulum Membrane-Induced Insulin Resistance. Front. Endocrinol..

[50] Szymanski J., Janikiewicz J., Michalska B., Patalas-Krawczyk P., Perrone M., Ziolkowski W., Duszynski J., Pinton P., Dobrzyn A., Wieckowski M.R. (2017). Interaction of Mitochondria with the Endoplasmic Reticulum and Plasma Membrane in Calcium Homeostasis, Lipid Trafficking and Mitochondrial Structure. Int. J. Mol. Sci..

[51] Weissmann G., Dukor P. (1970). The role of lysosomes in immune responses. Adv. Immunol..

[52] Davidson S.M., Vander Heiden M.G. (2017). Critical Functions of the Lysosome in Cancer Biology. Annu. Rev. Pharmacol. Toxicol..

[53] Ducatelle R., Hoorens J. (1984). Significance of lysosomes in the morphogenesis of coronaviruses. Arch. Virol..

[54] Andrews N.W. (2000). Regulated secretion of conventional lysosomes. Trends Cell Biol..

[55] Andrews N.W. (2005). Membrane repair and immunological danger. EMBO Rep..

[56] Chen D., Zheng Q., Sun L., Ji M., Li Y., Deng H., Zhang H. (2021). ORF3a of SARS-CoV-2 promotes lysosomal exocytosis-mediated viral egress. Dev. Cell.

[57] Guntuku L., Gangasani J.K., Thummuri D., Borkar R.M., Manavathi B., Ragampeta S., Vaidya J.R., Sistla R., Vegi N.G.M. (2019). IITZ-01, a novel potent lysosomotropic autophagy inhibitor, has single-agent antitumor efficacy in triple-negative breast cancer in vitro and in vivo. Oncogene.

[58] Mindell J.A. (2012). Lysosomal acidification mechanisms. Annu. Rev. Physiol..

[59] Motyka B., Korbutt G., Pinkoski M.J., Heibein J.A., Caputo A., Hobman M., Barry M., Shostak I., Sawchuk T., Holmes C.F. (2000). Mannose 6-phosphate/insulin-like growth factor II receptor is a death receptor for granzyme B during cytotoxic T cell-induced apoptosis. Cell.

[60] Reczek D., Schwake M., Schroder J., Hughes H., Blanz J., Jin X., Brondyk W., Van Patten S., Edmunds T., Saftig P. (2007). LIMP-2 is a receptor for lysosomal mannose-6-phosphate-independent targeting of beta-glucocerebrosidase. Cell.

[61] Scott C.C., Vacca F., Gruenberg J. (2014). Endosome maturation, transport and functions. Semin. Cell Dev. Biol..

[62] Chen Y., Yu L. (2013). Autophagic lysosome reformation. Exp. Cell Res..

[63] Gegg M.E., Schapira A.H. (2016). Mitochondrial dysfunction associated with glucocerebrosidase deficiency. Neurobiol. Dis..

[64] Yang Z., Goronzy J.J., Weyand C.M. (2015). Autophagy in autoimmune disease. J. Mol. Med..

[65] Ge W., Li D., Gao Y., Cao X. (2015). The Roles of Lysosomes in Inflammation and Autoimmune Diseases. Int. Rev. Immunol..

[66] Forrester S.J., Kikuchi D.S., Hernandes M.S., Xu Q., Griendling K.K. (2018). Reactive Oxygen Species in Metabolic and Inflammatory Signaling. Circ. Res..

[67] Wang X., Melino G., Shi Y. (2021). Actively or passively deacidified lysosomes push beta-coronavirus egress. Cell Death Dis..

[68] Westerbeck J.W., Machamer C.E. (2019). The Infectious Bronchitis Coronavirus Envelope Protein Alters Golgi pH To Protect the Spike Protein and Promote the Release of Infectious Virus. J. Virol..

[69] Ballabio A., Bonifacino J.S. (2020). Lysosomes as dynamic regulators of cell and organismal homeostasis. Nat. Rev. Mol. Cell Biol..

[70] Buratta S., Tancini B., Sagini K., Delo F., Chiaradia E., Urbanelli L., Emiliani C. (2020). Lysosomal Exocytosis, Exosome Release and Secretory Autophagy: The Autophagic- and Endo-Lysosomal Systems Go Extracellular. Int. J. Mol. Sci..

[71] Alesi N., Akl E.W., Khabibullin D., Liu H.J., Nidhiry A.S., Garner E.R., Filippakis H., Lam H.C., Shi W., Viswanathan S.R. (2021). TSC2 regulates lysosome biogenesis via a non-canonical RAGC and TFEB-dependent mechanism. Nat. Commun..

[72] Fujishima A., Imai Y., Nomura T., Fujisawa Y., Yamamoto Y., Sugawara T. (1997). The crystal structure of human cathepsin L complexed with E-64. FEBS Lett..

[73] Liu T., Luo S., Libby P., Shi G.P. (2020). Cathepsin L-selective inhibitors: A potentially promising treatment for COVID-19 patients. Pharmacol. Ther..

[74] Bode L., Dietrich D.E., Spannhuth C.W., Ludwig H. (2022). Prominent Efficacy of Amantadine against Human Borna Disease Virus Infection In Vitro and In Vivo. Comment on Fink et al. Amantadine Inhibits SARS-CoV-2 In Vitro. *Viruses*
**2021**, *13*, 539. Viruses.

[75] Cortes-Borra A., Aranda-Abreu G.E. (2021). Amantadine in the prevention of clinical symptoms caused by SARS-CoV-2. Pharmacol. Rep..

[76] Fink K., Nitsche A., Neumann M., Grossegesse M., Eisele K.H., Danysz W. (2021). Amantadine Inhibits SARS-CoV-2 In Vitro. Viruses.

[77] Yu F., Pan T., Huang F., Ying R., Liu J., Fan H., Zhang J., Liu W., Lin Y., Yuan Y. (2022). Glycopeptide Antibiotic Teicoplanin Inhibits Cell Entry of SARS-CoV-2 by Suppressing the Proteolytic Activity of Cathepsin L.. Front. Microbiol..

[78] Jacquin E., Leclerc-Mercier S., Judon C., Blanchard E., Fraitag S., Florey O. (2017). Pharmacological modulators of autophagy activate a parallel noncanonical pathway driving unconventional LC3 lipidation. Autophagy.

[79] Ahmadi A.R., Ayazi-Nasrabadi R. (2021). Astaxanthin protective barrier and its ability to improve the health in patients with COVID-19. Iran. J. Microbiol..

[80] Gomes C.P., Fernandes D.E., Casimiro F., da Mata G.F., Passos M.T., Varela P., Mastroianni-Kirsztajn G., Pesquero J.B. (2020). Cathepsin L in COVID-19: From Pharmacological Evidences to Genetics. Front. Cell Infect. Microbiol..

[81] Wang S.Q., Du Q.S., Zhao K., Li A.X., Wei D.Q., Chou K.C. (2007). Virtual screening for finding natural inhibitor against cathepsin-L for SARS therapy. Amino Acids.

[82] Kang S.N., Lee J.S., Park J.H., Cho J.H., Cho K.K., Lee O.H., Kim I.S. (2014). In vitro anti-osteoporosis properties of diverse Korean *Drynariae rhizoma* phenolic extracts. Nutrients.

[83] De Duve C., de Barsy T., Poole B., Trouet A., Tulkens P., Van Hoof F. (1974). Commentary. Lysosomotropic agents. Biochem. Pharmacol..

[84] Batiha G.E., Shaheen H.M., Al-Kuraishy H.M., Teibo J.O., Akinfe O.A., Al-Garbee A.I., Teibo T.K.A., Kabrah S.M. (2021). Possible mechanistic insights into iron homeostasis role of the action of 4-aminoquinolines (chloroquine/hydroxychloroquine) on COVID-19 (SARS-CoV-2) infection. Eur. Rev. Med. Pharmacol. Sci..

[85] Schrezenmeier E., Dorner T. (2020). Mechanisms of action of hydroxychloroquine and chloroquine: Implications for rheumatology. Nat. Rev. Rheumatol..

[86] Chen Y., Li M.X., Lu G.D., Shen H.M., Zhou J. (2021). Hydroxychloroquine/Chloroquine as Therapeutics for COVID-19: Truth under the Mystery. Int. J. Biol. Sci..

[87] Saghir S.A.M., AlGabri N.A., Alagawany M.M., Attia Y.A., Alyileili S.R., Elnesr S.S., Shafi M.E., Al-Shargi O.Y.A., Al-Balagi N., Alwajeeh A.S. (2021). Chloroquine and Hydroxychloroquine for the Prevention and Treatment of COVID-19: A Fiction, Hope or Hype? An Updated Review. Ther. Clin. Risk Manag..

[88] Blaess M., Kaiser L., Sommerfeld O., Rentschler S., Csuk R., Deigner H.P. (2020). Rational Drug Repurposing: Focus on Lysosomotropism, Targets in Disease Process, Drug Profile, and Pulmonary Tissue Accumulation in SARS-CoV-2 Infection/COVID-19. Front. Pharmacol..

[89] Gorshkov K., Chen C.Z., Bostwick R., Rasmussen L., Tran B.N., Cheng Y.S., Xu M., Pradhan M., Henderson M., Zhu W. (2021). The SARS-CoV-2 Cytopathic Effect Is Blocked by Lysosome Alkalizing Small Molecules. ACS Infect. Dis..

[90] Meneses Calderon J., Figueroa Flores M.D.R., Paniagua Coria L., Briones Garduno J.C., Meneses Figueroa J., Vargas Contretas M.J., De la Cruz Avila L., Diaz Meza S., Ramirez Chacon R., Padmanabhan S. (2020). Nitazoxanide against COVID-19 in three explorative scenarios. J. Infect. Dev. Ctries..

[91] Chiappori A., Williams C., Northfelt D.W., Adams J.W., Malik S., Edelman M.J., Rosen P., Van Echo D.A., Berger M.S., Haura E.B. (2014). Obatoclax mesylate, a pan-bcl-2 inhibitor, in combination with docetaxel in a phase 1/2 trial in relapsed non-small-cell lung cancer. J. Thorac. Oncol..

[92] Wang R., Wang J., Hassan A., Lee C.H., Xie X.S., Li X. (2021). Molecular basis of V-ATPase inhibition by bafilomycin A1. Nat. Commun..

[93] Hart P.D., Young M.R. (1991). Ammonium chloride, an inhibitor of phagosome-lysosome fusion in macrophages, concurrently induces phagosome-endosome fusion, and opens a novel pathway: Studies of a pathogenic mycobacterium and a nonpathogenic yeast. J. Exp. Med..

[94] Dabydeen S.A., Meneses P.I. (2009). The role of NH4Cl and cysteine proteases in Human Papillomavirus type 16 infection. Virol. J..

[95] Shang C., Zhuang X., Zhang H., Li Y., Zhu Y., Lu J., Ge C., Cong J., Li T., Tian M. (2021). Inhibitors of endosomal acidification suppress SARS-CoV-2 replication and relieve viral pneumonia in hACE2 transgenic mice. Virol. J..

[96] Zeng J., Shirihai O.S., Grinstaff M.W. (2019). Degradable Nanoparticles Restore Lysosomal pH and Autophagic Flux in Lipotoxic Pancreatic Beta Cells. Adv. Healthc. Mater..

[97] Cunha A., Gaubert A., Latxague L., Dehay B. (2021). PLGA-Based Nanoparticles for Neuroprotective Drug Delivery in Neurodegenerative Diseases. Pharmaceutics.

[98] Yang M., Jin L., Wu Z., Xie Y., Zhang P., Wang Q., Yan S., Chen B., Liang H., Naman C.B. (2021). PLGA-PEG Nanoparticles Facilitate In Vivo Anti-Alzheimer’s Effects of Fucoxanthin, a Marine Carotenoid Derived from Edible Brown Algae. J. Agric. Food Chem..

[99] Pourhajibagher M., Azimi M., Haddadi-Asl V., Ahmadi H., Gholamzad M., Ghorbanpour S., Bahador A. (2021). Robust antimicrobial pho todynamic therapy with curcumin-poly (lactic-co-glycolic acid) nanoparticles against COVID-19: A preliminary in vitro study in Vero cell line as a model. Photodiagnosis Photodyn. Ther..

