# Supplementary material for: Potential Antiviral Strategy Exploiting Dependence of SARS-CoV-2 Replication on Lysosome-Based Pathway
Source: Int J Mol Sci. 2022 May 31;23(11):6188. doi: 10.3390/ijms23116188 (PMC9181800; doi:10.3390/ijms23116188)
Supplement: Supplementary file 1 [file ijms-23-06188-s001.zip › ijms-1739898-supplementary.pdf]

Supplementary File S1

Table S1. The antiviral effect of the biomaterials within the manuscript.

Biomaterials	Modulating lysosomal function	Anti-SARS-CoV-2 effect	Reference
Amantadine	Inhibiting CTSL activity	++	[74-76]
Teicoplanin	Inhibiting CTSL activity	+	[77]
Astaxanthin	Inhibiting CTSL activity	++	[78, 79]
MOL736	Inhibiting CTSL activity	+	[81]
Water and ethanol extracts of Drynariae Rhizoma	Inhibiting CTSL activity	−	[73, 82]
Choloroquine	Rasing the lysosomal PH	++	[84, 85]
Hydroxychloroquine	Rasing the lysosomal PH	++	[84, 85]
ROC-325	Rasing the lysosomal PH	+	[89]
Nitazoxanide	Rasing the lysosomal PH	++	[90]
IITZ-01	Rasing the lysosomal PH	−	[57]
IITZ-02	Rasing the lysosomal PH	−	[57]
Obatoclax mesylate	Rasing the lysosomal PH	+	[91]
Bafilomycin A1	Rasing the lysosomal PH	++	[95]
NH_4Cl	Rasing the lysosomal PH	++	[95]
PLGA	Lowering the lysosomal PH	−	[96]

++ Suppressing viral infection *in vitro* and *in vivo*; + Suppressing viral infection *in vitro*; − Unknown.
